# Inter-individual variability (but intra-individual stability) of overt versus covert rehearsal strategies in a digital Corsi task

**DOI:** 10.1167/jov.24.8.2

**Published:** 2024-08-01

**Authors:** Lílian de Sardenberg Schmid, Gregor Hardiess

**Affiliations:** 1 Cognitive Neuroscience, Department of Biology, Institute of Neurobiology, University of Tübingen, Tübingen, Germany; 2 Systems Neuroscience & Neuroengineering, MPI for Biological Cybernetics, Tübingen, Germany

**Keywords:** visuospatial working memory, Corsi block-tapping task, Corsi span, eye movements, covert attention, overt attention, rehearsal, pupil size

## Abstract

The Corsi (block-tapping) paradigm is a classic and well-established visuospatial working memory task in humans involving internal computations (memorizing of item sequences, organizing and updating the memorandum, and recall processes), as well as both overt and covert shifts of attention to facilitate rehearsal, serving to maintain the Corsi sequences during the retention phase. Here, we introduce a novel digital version of a Corsi task in which i) the difficulty of the memorandum (using sequence lengths ranging from 3 to 8) was controlled, ii) the execution of overt and/or covert attention as well as the visuospatial working memory load during the retention phase was manipulated, and iii) shifts of attention were quantified in all experimental phases. With this, we present behavioral data that demonstrate, characterize, and classify the individual effects of overt and covert strategies used as a means of encoding and rehearsal. In a full within-subject design, we tested 28 participants who had to solve three different Corsi conditions. While in condition A neither of the two strategies were restricted, in condition B the overt and in condition C the overt as well as the covert strategies were suppressed. Analyzing Corsi span, (eye) exploration index, and pupil size (change), data clearly show a continuum between overt and covert strategies over all participants (indicating inter-individual variability). Further, all participants showed stable strategy choice (indicating intra-individual stability), meaning that the preferred strategy was maintained in all three conditions, phases, and sequence lengths of the experiment.

## Introduction

Many daily activities, such as selecting items in the kitchen to prepare food, require the encoding, maintenance, and retrieval of spatiotemporally distributed stimuli. In the present study, the so-called Corsi block-tapping task (Corsi, 1972), in which a sequence of taps on identical but spatially distributed blocks has to be memorized and reproduced, is used to investigate the handling and processing of spatiotemporally distributed stimuli in greater detail under experimental conditions. In the course of such a task, participants have to variably recruit and allocate memory, pay attention, and plan motor behavior to resolve the conflict appropriately between the different task demands on the one hand but also their own preferences regarding the required processes on the other (Droll & Hayhoe, 2007; Draschkow, Kallmayer, & Nobre 2021; Hardiess & Mallot, 2015). The overall aim of this study was to test a cohort of participants in a Corsi task, systematically recording and monitoring eye movements to investigate and classify the individual effects of overt and covert strategies used as a means of encoding and rehearsal of spatiotemporally distributed stimuli. A more detailed description of the objectives and research questions of this study can be found in the Plan of the paper.

### The trinity in visuospatial processing

Three functions are central in the course of visuospatial processing, namely, i) visuospatial working memory (VSWM), ii) visuospatial attention, and iii) the oculomotor control (see Boon, Theeuwes, & Belopolsky, 2019; Heuer, Ohl, & Rolfs, 2020; Olivers & Roelfsema, 2020; Smith & Archibald, 2020). These three functions are closely inter-connected, both in the way they operate and in the way they have been investigated and defined over the last decades (Theeuwes, Belopolsky, & Olivers, 2009; Unsworth & Robison, 2017; Williams, Pouget, Boucher, & Woodman, 2013).

VSWM is a fundamental, functional component of cognition (Baddeley & Hitch, 1974; Cowan, 2017; Eriksson, Vogel, Lansner, Bergström, & Nyberg, 2015) and represents and processes information over seconds to minutes in a way that allows the agent to behave beyond the here and now (Christophel, Klink, Spitzer, Roelfsema, & Haynes, 2017; Zimmer, 2008). However, VSWM is strictly limited in capacity (e.g., Cowan, 2010; Luck & Vogel, 2013) and therefore needs active maintenance and on-line manipulation of task-relevant information (Masse, Yang, Song, Wang, & Freedman, 2019). The use of attention is a proposed mechanism serving to protect the memorandum from interfering with internal or external distractions and temporal decay (Awh & Jonides, 2001; Baddeley, 2012; Farrell et al., 2016). The VSWM is tightly coupled with the neural and cognitive processes involved in visuospatial attention and oculomotor control (Awh & Jonides, 2001; Awh, Vogel, & Oh, 2006; Heuer et al., 2020; Theeuwes et al., 2009). Attention is the process of selecting or prioritizing objects or stimuli from the external or internal “world” (Christophel, Iamshchinina, Yan, Allefeld, & Haynes, 2018; Myers, Stokes, & Nobre, 2017). Because humans rely on foveated vision, they depend on the execution of eye movements to bring regions of interest from the periphery into their central field of view. In the oculomotor system, eye movements are controlled by VSWM as well as attention to sample and select items from the environment to transfer their task-relevant information into the perceptual system (Schütz-Bosbach & Prinz, 2007; van Ede, 2020). The target selection for both attention and eye movement can vary between a rather automatic and stimulus-driven (bottom–up) and a cognitive and task-driven (top–down) process. Owing to the tight coupling with the VSWM and attention, eye movements can also support mental processes responsible for the retention and retrieval of stimuli (i.e., rehearsal, imagery) should visual input be costly, uninformative or absent (Brandt & Stark, 1997; Lilienthal, Myerson, Abrams, & Hale, 2018; Souza, Czoschke, & Lange, 2020; Zimmer, 2008).

### Overt versus covert shifts in visuospatial attention

Visuospatial selection is an active process (O'regan & Noë, 2001) that can be decoupled from eye or gaze movements (Boon et al., 2019). During overt shifts of focal attention, the eyes move and are directed (by means of fixational movements) directly to the object(s) of interest (Bisley, 2011; Krauzlis, Lovejoy, & Zénon, 2013; Posner, 1980). Here, it does not matter whether the object is moving or stationary. During overt attention, the visual reference (frame) for motor control centers on the fixation point (Boon et al., 2019; Zhou, Liang, Pan, Qian, & Zhang, 2017) and overt orientation can be directly observed and measured with an eye tracker (e.g., Hardiess, Gillner, & Mallot, 2008). Covert shifts of attention play a role when the eyes do not move (e.g., perform a stable fixation), but the focus of attention shifts across the field of view, i.e., to the periphery (Bisley, 2011; Fehd & Seiffert, 2008; Hardiess, Hansmann-Roth, & Mallot, 2013; Moore, Armstrong, & Fallah, 2003; Posner, 1980).

Within the framework of premotor theory, covert attention is a consequence of the preparation of eye movements, that is, a byproduct of saccade planning and not per se limited to the eye movement system (Rizzolatti, Riggio, Dascola, & Umiltá, 1987; Smith & Schenk, 2012). The visual attention model argues that both covert attention and motor preparation are the consequence of the same selection mechanism (Deubel & Schneider, 1996; Schneider, 1995). In such a selection, an object of interest is prioritized and the two processes, the enhancement of the processing of its features (selection for perception) as well as the preparation of a motor action towards it (selection for action), are activated. In both theories, covert attention and action preparation are coupled tightly. This view is supported by the findings that the same brain areas seem to be involved in both attention and eye movement preparation (e.g., Perry & Zeki, 2000; Silvanto, Lavie, & Walsh, 2006). Recent and more comprehensive approaches extend the above theories by attempting to link the processes of attention and motor actions with the functions of VSWM (Heuer et al., 2020; Olivers & Roelfsema, 2020). For a more detailed description of the various theories, please read the Discussion section (see Discussing rehearsal strategies).

### The Corsi paradigm

VSWM enables, among others, the temporary maintenance and processing of visuospatial information and the allocation of resources to its acquisition and use on several stages of cognitive processing (Eriksson et al., 2015; Fuster & Bressler, 2012). Therefore, VSWM is essential to perform visuospatial tasks ranging from simply memorizing positions of external items for later usage (e.g., Pearson & Sahraie, 2003) over wayfinding through to spatial planning and problem solving (Hambrick & Engle, 2003; Kravitz, Saleem, Baker, & Mishkin, 2011; Loomis, Klatzky, & Giudice, 2013). A classic paradigm for studying VSWM in clinical examination and basic research is the Corsi (block-tapping) task developed by Philip Corsi (Corsi, 1972; Milner, 1971). In the original version, participants observed a sequence of taps on a set of identical wooden blocks distributed on a table that they then had to reproduce. In general, spatiotemporally distributed visual items have to be encoded and maintained (for seconds) to reproduce the relevant Corsi sequence correctly afterward in a recall phase. During the task, Corsi sequences increase in length (and complexity). The Corsi span (i.e., the sequence length or complexity where the subject's performance breaks down) as well as other VSWM-related measures can be analyzed together with eye movements to quantify spatial memory and to investigate the attentional strategies applied by the participants (e.g., Conway et al., 2005; Pearson & Sahraie, 2003; Röser, Hardiess, & Mallot, 2016). In digital versions of the Corsi task, the sequence is displayed on a screen and participants reproduce it by clicking with a mouse. This strategy allows for reliable eye-tracking throughout all phases of the experiment and full control over confounds like the geometrical complexity of the to-be-remembered sequence (Orsini, Pasquadibisceglie, Picone, & Tortora, 2001) or discrepancies in the motor effort associated with encoding versus reproduction modalities (Röser et al., 2016).

Overt and covert attention (shifts) play a significant role to form a serial and spatial memory during the encoding phase of the Corsi task, and in the retention phase rehearsal processes are needed to maintain information actively (Awh et al., 2006; Godijn & Theeuwes, 2012; Pearson & Sahraie, 2003; Unsworth & Robison, 2018). There are essentially two plausible mechanisms that support rehearsal. In the attention-based mechanism (Awh, Jonides, & Reuter-Lorenz, 1998; Lilienthal et al., 2018), rehearsal is triggered exogenously by the stimulus itself, whereas in the mental-based mechanism, mental rehearsal (Cisek & Kalaska, 2004) is initiated endogenously, requiring imagery processes (Pearson, 2019). Here, participants imagine the relevant visuospatial features in front of the mind's eye (Kosslyn, Ganis, & Thompson, 2001). The present study focuses on the attention-based mechanism. To minimize the use of imagery processes by the participants, they were required to keep their eyes open at all times during each phase of the experiment. This was controlled by eye tracking. Furthermore, during the experimental retention phase, participants had to attend to secondary-task stimuli, requiring their attention to be directed externally (Villena-González, López, & Rodríguez, 2016).

Both covert and overt attention shifts can be used for attention-based rehearsal to counteract time-based forgetting (Godijn & Theeuwes, 2012; Lilienthal et al., 2018; Souza et al., 2020). There is clear evidence that overt oculomotor rehearsal is beneficial for maintenance of the sequential information in Corsi tasks (i.e., Baddeley, 1986; Tremblay, Saint-Aubin, & Jalbert, 2006; see also Unsworth & Robison [2017] for more citations). In the context of the Corsi task, the benefit of retracing the to-be-remembered sequence with eye movements might be explained with the outsourcing of the spatiotemporal information maintenance onto the executed eye movements, freeing cognitive resources (Boon et al., 2019). In contrast, a substantial amount of studies report covert shifts of attention as means to rehearse the spatial order or the pattern of Corsi sequences (i.e., Patt et al., 2014; Godijn & Theeuwes, 2012). Covertly encoding and rehearsing a visuospatial sequence as a sequence of saccadic motor plans could benefit the maintenance in a similar way as retracing with actual execution of eye movements does (Pearson, Ball, & Smith, 2014).

Given the various studies examining overt and/or covert attention in a Corsi paradigm, it is not clear from the literature whether overt and covert rehearsal are similarly effective in maintaining VSWM. Given the choice between overt and covert shifts of attention, it is also unclear whether participants use both options depending on the task complexity (intra-individual switching,) or whether they limit themselves to one of the two options across all trials.

### Pupillary light reflex (PLR) as a means to detect covert attention shifts

One way to detect covert shifts of attention in the absence of eye movements is to make use of the PLR. Even with the eyes fixated on a neutral point, pupils will dilate or constrict in the range of millimeters when visual attention is covertly shifted to a darker (dilation) or brighter (constriction) area, respectively (Joshi & Gold, 2020; Unsworth & Robinson, 2017). In addition, the manageable sequence length in the context of a Corsi task, that is, the cognitive or mental load, has also been shown to have an effect on pupil size (van der Wel & Van Steenbergen, 2018; Tsukahara & Engle, 2021) that is expected to be independent of the applied rehearsal strategy. Cognitive load causes the pupil to dilate, that is, the pupil widens in the range of less than 1 mm (Tsukahara & Engle, 2021). Here, pupil dilation refers to the stimulus-induced increase in pupil diameter relative to a prestimulus baseline period. By controlled manipulation of the background illumination during (sub)trials with constant cognitive load (i.e., the same sequence length), it should be possible to differentiate between the rehearsal strategies based on the magnitude and direction of the change in pupil size (Hu, Hisakata, & Kaneko, 2022; Mathôt, 2018). Therefore, the combination of eye-tracking technology (which allows recording of both eye position and pupil size) with a digital version of the Corsi task is ideal to cope with such strategy disambiguation.

### Plan of the paper

There is evidence that both overt and covert attention-based as well as mental rehearsal strategies are used in the maintenance of visuospatial sequences. To prevent internalized mental rehearsal throughout the experiment, our participants had to keep their eyes open and had to attend to secondary-task stimuli during retention. We focus our experimental design and analysis on disambiguating between the two attention-based rehearsal strategies, overt versus covert. Overt attention shifts can be detected with an eye tracker through the associated eye movements. We were also able to quantify the magnitude of overt attention using the exploration index, that is, taking into account the extent of eye movements toward the Corsi items. By instrumentalizing the PLR in our experimental design, covert shifts of attention can be detected by changes in pupil size. As we manipulate the difficulty of the task (increasing sequence length and complexity) and change the background illumination for each sequence length between black and white subtrials, we can use the extent and direction of pupil change to distinguish between the two rehearsal strategies.

Despite several studies reporting the role of gaze movements as means regarding rehearsal in VSWM tasks, no one has investigated and quantified the inter-individual variability of such oculomotor rehearsal mechanism measuring eye movements in all three phases (encoding, maintenance, and recall) of a Corsi task so far. In a full within-subject design, we tested a random sample of participants who had to solve three different Corsi conditions, with eye movements and pupil size recorded throughout the experiment. While in condition A neither of the two rehearsal strategies were restricted, in condition B the overt and in condition C the overt as well as the covert strategy were suppressed with a secondary task requiring VSWM resources. In all conditions, phases, and sequence lengths of the experiment, the background illumination changed systematically to measure meaningful changes in pupil size. Thus, we were able to control for overt versus covert shifts of attention together with cognitive load in each subtrial. We then will analyze the measures Corsi span, (eye) exploration index, and pupil size (change), to answer the following questions. First, have individual participants a fixed preference for either rehearsal strategy or do they switch randomly or strategically dependent on sequence properties? And, second, does one of the two strategies offer a performance benefit in the context of the Corsi task? With this investigation, we are able to i) classify the participants into a visuospatial retention strategy, ii) test the robustness of such individual strategies over different lengths of Corsi sequences and over the Corsi phases, as well as conditions, and iii) quantify the benefit of the chosen strategy when manipulating the particular use of it.

The initial idea of our study design was to completely prevent the execution of eye movements in the retention phase of condition B. Participants who preferred overt rehearsal would, therefore, either not have been able to rehearse in this phase or would have had to switch to the covert strategy. However, the results of the study show that the participants who prefer overt rehearsal performed small-scaled but meaningful eye movements within a window of tolerance during the retention phase of condition B. Such a tolerance window was defined, because the gaze or its measurement could be unstable (see Experimental conditions). In subtrials with a white background, such eye movements cause the pupil to constrict more owing to the PLR than would be the case with covert shifts of attention. We used this difference to our advantage in the later analysis to distinguish between overt and covert rehearsal strategies.

## Material and methods

### Ethics statement

All aspects of the experiment were approved by the Ethics Committee of the Faculty of Psychology of the University of Tübingen (number of approval: Hardiess_2018_ 0905_134, 17.10.2018) and conformed to the Declaration of Helsinki for testing human participants. All participants read and signed a written informed consent before taking part in the study.

### Participants

In total, 35 volunteers (students from Tübingen University) were invited and participated in the study. Four of them were excluded because of technical insufficiencies concerning eye-tracking. Another three participant were quantified as outliers, because their Corsi span never exceeded a value of nine, that is, they could not correctly perform a sequence length above four. Finally, 28 participants were investigated (11 male; aged between 18 and 30 years old, *M* = 22.5, *SD* = 3.52). All participants were naïve to the purpose of the experiment, had normal or corrected-to-normal vision, were right handed, and received either course credit or a monetary compensation (10€ per hour).

### Apparatus

The experiment was conducted on a personal computer (3.1 GHz) using a Samsung SyncMaster 931BF monitor (19’’, 1280 × 1024 pixel, ±20.6 x ±16.7°, 60 Hz). The viewing distance between participant and monitor was 60 cm (chin rest used) and the stimuli were viewed in a dimly lit and noise-reduced room. MATLAB 2017b (MathWorks, Natick, MA) together with the Psychophysics Toolbox-3 extension (Brainard, 1997; Kleiner et al., 2007) were used for stimulus presentation, experiment control, and recording of eye position, as well as participants’ responses. Eye movements were tracked and recorded throughout the experiment in a non-invasive way using a monocular, remote, and infrared light-based eye tracker (EyeGaze system, LC Technology, Inc., Salisbury, MA; and IMD GmbH, Hanover, Germany) with a sampling rate of 60 Hz. The quality of tracking was high for all participants, that is, the overall loss of tracking data was approximately 4% (*µ* = 0.039, *σ* = 0.15, *m* = 0.0) with no significant difference between three different conditions.

### Stimuli

**Figure 1.**
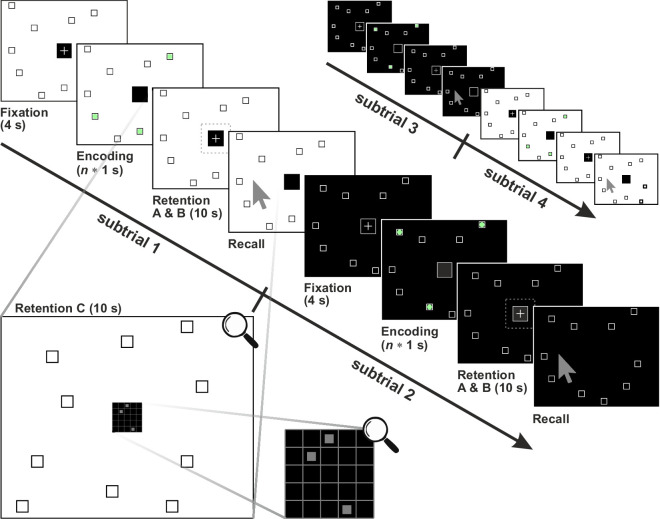
Experimental paradigm, trial setup and conditions exemplary shown for the sequence length 3. Each trial consists of four subtrials with an order of background color of white, black, black, white. Each subtrial uses a different default pattern randomly chosen out of five templates. A subtrial consists of a 4-second lasting fixation phase, a *n* times 1-second (*n* = sequence length) lasting encoding phase, a 10-second lasting retention phase, and a recall phase. Targets of the Corsi sequence are displayed with green (RGB color: 153|255|153) circles (please note that all target are displayed here simultaneously). The area of the fixation window (black square) in the fixation phase is 150 × 150 pixels and the tolerance window (dashed square) in the retention phase is 200 × 200 pixels. A possible secondary task display of the condition C (restricted overt and covert strategies) of the retention phase is shown enlarged in the lower part of the figure. Note, the fixation cross was shown during the fixation phase (all conditions) and in the retention phase (only condition B; i.e., restricted overt strategy).

All Corsi sequences used in the study were generated basing on five different default patterns (Supplementary Figure S1). These patterns (and the sequences) were randomly initiated and manually checked and corrected to balance their distribution/complexity. Each pattern consisted of 10 squares (size of one square: 60 × 60 pixels, 2 × 2°). The 10 squares of a default pattern were evenly distributed around the central fixation window (size of the fixation window: 150 × 150 pixels, 5 × 5°; cf., Figure 1) and the overall spatiotemporal complexity between the 10 squares and the five patterns was balanced concerning the number of path crossings, path lengths, and path angles (Busch, Farrell, Lisdahl-Medina, & Krikorian, 2005; Parmentier, Elford, & Maybery, 2005). For the two different backgrounds (white and black), the squares were outlined in different shades of gray (light gray [RGB color: 210|210|210] for white and dark gray [RGB color: 45|45|45] for black background), leading to a similar perceived contrast between them and the background. The values of gray tones were determined by asking former participants about their perceived contrasts in a test before programming the experiment. For each subtrial, one of the five default patterns was pseudo-randomly chosen, defining the selectable positions for the corresponding Corsi sequence. For each experimental condition and Corsi sequence length, a participant had to perform one trial consisting of four subtrials. To vary the difficulty of the Corsi task over the experiment, sequence length started for each condition with 3 and ranged up to 8 (progressively increasing in length). The needed squares for a respective sequence length were pseudo-randomly chosen from the actual default pattern to minimize the so called path length effect (i.e., varying spatial lengths of Corsi sequences with the same sequence length can affect the Corsi span as a confounding variable; De Lillo, Kirby, & Poole, 2016). In addition, Corsi sequences with the same sequence length covered a maximum of the screen and had similar numbers of path crossings.

### Corsi task and experimental conditions

#### Corsi task

As with the classic Corsi task, each individual subtrial in this study always comprised four phases (Figure 1). During the 4-second lasting initial fixation phase, a participant was instructed to fixate continuously the cross in the middle of the screen. This phase was important to prepare the participant for the current subtrial and to measure the stabilized pupil size needed later for the method of baseline correction (see Data analysis and dependent variables). Besides the cross, also the 10 squares of the default pattern were displayed in the fixation phase. Immediately after fixation, the encoding phase started. Here, the current Corsi sequence was presented to the participant with the task to encode (memorize) it for later recall. The spatiotemporal sequence was presented by highlighting the targets of the current sequence one after another for one second each (interstimulus interval between items was 0 second) using a green circle filling up a square (RGB color: 153|255|153). The duration of this phase depended on the number of targets and lasted from 3 seconds (sequence length = 3) to 8 seconds (sequence length = 8). The most important phase for the purpose of the study was the next, namely the retention phase. This phase lasted always 10 seconds and participants were instructed to maintain the encoded Corsi sequence as precisely as possible while performing different secondary tasks (the kind of secondary tasks was defined by condition). The 10 squares of the default pattern were displayed during the entire retention phase. The final phase of a subtrial was the recall phase. Here, presenting the default squares together with a mouse pointer, a participant should click into the target-squares of the remembered Corsi sequence in the correct order one after another. If a click was correct in terms of location and temporal order, a green circle (RGB color: 153|255|153) was displayed in the chosen square for 1 second, if not, a red circle (RGB color: 255|102|102) appeared. If the participant clicked *n* times (*n* = sequence length), the recall phase ended and the next subtrial started with the fixation phase.

#### Experimental conditions

To investigate the overt and covert attention strategies applied by the participants to rehearse and maintain the Corsi sequences during the retention phase, three different conditions were tested in this study (cf., Figure 1).

##### Condition A: Without any secondary task

Here, participants were not instructed to perform a secondary task and were, therefore, free to choose a convenient overt or covert strategy for maintenance.

##### Condition B: Restricted overt strategy

The objective for this condition was to prevent the use of overt gaze shifts during the retention phase as a strategy for rehearsal. Participants were instructed to fixate the fixation cross, that is, to keep their gaze within the fixation window for the complete duration of the retention phase. Because the gaze or its measurements could be unstable, a window of tolerance was defined around the fixation window (size of the tolerance window: 200 × 200 pixel, 6.7 × 6.7°). Please note that, despite the instruction to avoid any eye movements, some participants showed overt rehearsal within the window of tolerance. If the gaze left this tolerance window, the current subtrial was aborted immediately, a message appeared telling the participant to maintain their gaze on the fixation cross, and the subtrial was repeated immediately using a new Corsi sequence. Across all subtrials of condition B participants failed to maintain fixation within the tolerance window on average 3.4 times (σ = 3.04).

##### Condition C: Restricted overt and covert strategies

The objective of this condition was to prevent overt as well as covert attentional shifts during the retention phase. Therefore, a 5 × 5 grid was displayed in the center of the screen, that is, the secondary task window (size of the secondary task window: 150 × 150 pixel, 5 × 5°). After a random onset delay of a maximum of 2 seconds, 3 of the 25 randomly chosen grid cells were marked for 2 seconds (Figure 1). Here, the positions of markers were selected in a way that they did not cluster spatially. After a delay of 1.5 seconds (after marker disappearance), the participant should click with the mouse pointer into the previously marked cells in any order. The error rate was recorded and, if more than one error occurred, the participant was instructed to be more accurate at the end of the subtrial.

Condition C was introduced to show that the covert rehearsal strategy is used for type II participants (Corsi span mainly affected by condition C, preference for a covert strategy). To suppress the execution of covert attention, it is common to use a secondary or dual task to increase the demand to engage visuospatial attention to the screen center (e.g., see Souza et al., 2020).

### Overall task procedure

After a welcome, a participant had to read, understand, and sign the written informed consent, as well as the experimental instruction. Next, it was quantified if the participants’ gaze could be recorded properly. In the following, one test trial (including four subtrials) for each condition were conducted with sequence length 2 in a training block. After clarifying potentially remaining questions, the main experiment started. Here, each participant had to perform three experimental blocks (trial order: first block, A3–C3–B3–C4–B4–A4; second block, B5–A5–C5–A6–C6–B6; and third block, C7–B7–A7–B8–A8–C8; letters denote conditions and numbers sequence length). As explained elsewhere in this article, each trial (e.g., A3) contained four subtrials. Altogether, all participants were tested in a complete within-subject design performing a maximum amount of 18 trials (i.e., 72 subtrials) each. If a participant failed in more than two of the four subtrials of a certain trial, the respective condition was excluded in the following sequence length within a block. In total, the experiment took approximately 1 hour per participant. In the beginning of the presentation of a trial, the respective condition as well as sequence length was displayed for 1 second in the middle of the screen. The background color changed from subtrial 1 to 4 in the order white, black, black, white (Figure 1) to enable the analysis of covert shifts of attention using pupil size changes (see Data analysis and dependent variables). Between the three blocks, participants could take a break as they wished and the eye tracker was recalibrated again to ensure optimal tracking.

### Data analysis and dependent variables

For the scope of this study, three main dependent variables were calculated, namely, the Corsi span, exploration index, and pupil size change. Whereas the Corsi span includes all measured sequence lengths, for the variables exploration index and pupil size change just data from sequence lengths of 3 to 5 were used since participants showed an overall adequate performance in recalling the Corsi sequences only for these sequence lengths (Supplementary Table S1).

#### Corsi span

This measure characterizes the VSWM capacity and quantifies the level of Corsi task difficulty at which a participant was still able to perform the task correctly. The Corsi span is defined typically as the longest sequence that a participant can repeat correctly. For our digital Corsi task with four subtrials per sequence length, the Corsi span was calculated using the following formula: Corsi span = ($\sum_{SL=1}^{8}\#subtrials_{corr}\left(SL\right)*SL$)/4. Here, the number of correct subtrials per sequence length was weighted with the respective sequence length, added up for sequence length 1 to 8, and the final sum was divided by 4 (the number of subtrials). Sequence lengths 1 and 2 were not tested because they were too simple and here all four subtrials enter in Corsi span as correct ones. Using this calculation, the Corsi span could range between 3 and 36. The absolute Corsi span and its reduction between conditions were used to classify two types of participants, that is, type I (Corsi span mainly affected by condition B, a preference for an overt strategy) and type II (Corsi span mainly affected by condition C, a preference for a covert strategy).

#### Exploration index

**Figure 2.**
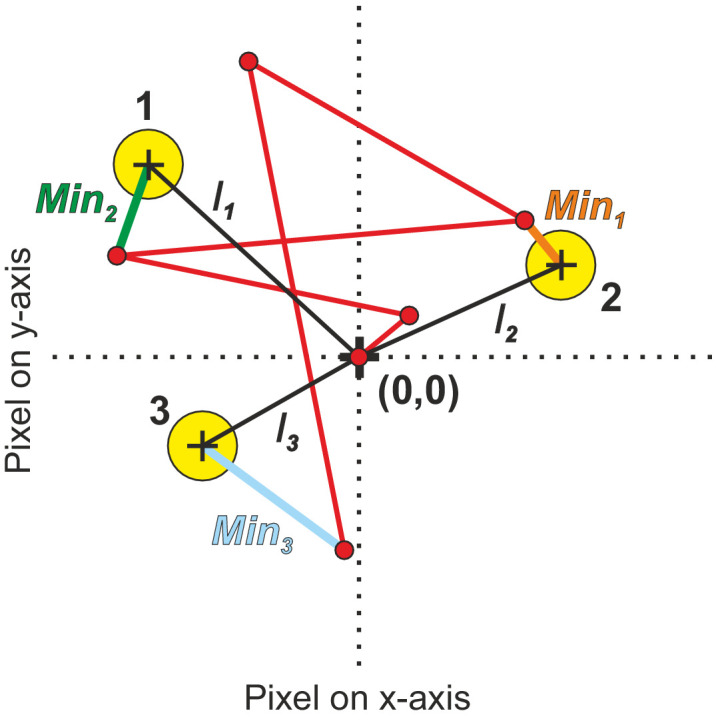
Scheme to illustrate the elements defining the two variables (*Dist_max_* = $\sum_{n=1}^{SL}\left(I_{n}\right)$ and *Dist_real_* = $\sum_{n=1}^{SL}\left(Min_{n}\right)$ needed for the calculation of exploration index for sequence length (SL) 3. The center of the Euclidean coordinate system is in the middle of the stimulus screen (0,0). *I_1_*, *I_2_*, and *I_3_* are the Euclidean distances (black lines) of the target positions (yellow circles with numbers) to the center. The red lines represent the gaze trajectory where fixations are depicted by red dots. *Min_1_*, *Min_2_*, and *Min_3_* are the Euclidean distances (orange, green, and blue lines) of the three fixations (closest to the targets) to the center of the corresponding targets.

To quantify the extent of overt attention (i.e., looking at items/targets) during the encoding and the retention phase, first fixations had to be calculated and then related to the positions of the targets of the Corsi sequences. To extract fixations, a velocity-based algorithm was used: For each time step *t_0_*, a gliding window of 120 ms length centered at *t_0_* was considered. Let *v_min_* and *v_max_* denote minimal and maximal eye velocities obtained within the window, respectively. The instant *t_0_* is classified as belonging to a fixation if *v_max_* − *v_min_* < 50 deg/s. This procedure is iterated through all time steps. Adjacent instants in time satisfying the condition are combined to fixational events (Figure 4). Two variables (Figure 2) were needed to calculate the exploration index: *Dist_max_* = $\sum_{n=1}^{SL}\left(I_{n}\right)$ and *Dist_real_* = $\sum_{n=1}^{SL}\left(Min_{n}\right)$. *Dist_max_* is the sum of the Euclidean distances of the target positions to the center (*I_n_*) for a given Corsi sequence. *Dist_real_* is the sum of the Euclidean distances of the *n* fixations (*n* = sequence length) to the corresponding targets (*Min_n_*) for a given Corsi sequence. If the number of fixations exceeded the number of targets, the *n* fixations closest to the target positions were selected. Now, a normalized exploration index could be calculated with: exploration index = 1 − *Dist_real_*/*Dist_max_*. The index ranged from 0 (i.e., all *n* fixations were located at the center) to 1 (i.e., the positions of the *n* fixations were identical with the targets). Again, an exploration index of 0 would be calculated if the sum of the distances between targets and their closest fixation is equal to the sum of the distances between targets and the center. Theoretically, an index of 0 could, therefore, also occur if the fixations were not located at the center. However, this possibility can be ruled out for our data, because we have checked the scanpaths for all participants with an exploration index of 0 and close to 0. Please also note that the exploration index only takes into account the spatial positions, but not the temporal order of these positions in the Corsi sequences.

#### Pupil size change

To calculate the pupil size change during the retention phase of condition B, we followed the procedure described by Unsworth and Robison (2017). First, pupil size data were baseline corrected by subtracting out the averaged baseline pupil diameter estimated during the last 30 frames (0.5 second) of the fixation phase on a subtrial-by-subtrial basis for each participant. Next, baseline corrected pupil data were averaged into a series of 100-ms time windows for each subtrial to smooth the traces. In the next step, the respective slope of the corrected and smoothed traces over the complete 600 frames (10 seconds) retention interval was calculated and the two slopes of the subtrials 1 and 4 with white background (and black center) as well as of the subtrials 2 and 3 with the black background were averaged.

We used black background subtrials (black) to quantify exclusively the cognitive load caused by the complexity of the task (i.e., maintaining the items with different sequence lengths). Because black only quantifies the cognitive load without any impact of rehearsal, solely the sequence length or task complexity influences the traces here. Higher load is associated with larger pupil size (with changes in the range of less than one millimeter), and increasing dilation during the 10-second retention phase can be quantified by a positive slope (Figure 7, black lines). Here, the pupil size cannot change owing to different light conditions, that is, the PLR does not affect pupil size in either overt or covert shifts of attention. The PLR is the rapid and relatively strong constriction/dilation of the pupil diameter in response to an increase or decrease in light intensity (Clarke, Zhang, & Gamlin, 2003; Joshi & Gold, 2020; Loewenfeld & Lowenstein, 1993). Subtrials with a white background but a black center (i.e., white; Figure 1) were used to measure pupil size under the influence of cognitive load together with the PLR. Here, the change of pupil size can be used to discriminate between oculomotor strategies. When participants maintain the overt strategy, the pupil constricted more than when a covert participant fixated strictly on the fixation cross that was presented on a dark background (Figure 1). When executing (small-scaled) eye movements, the white area surrounding the black center affects the constriction directly via the PLR resulting in large negative slopes (Figure 7C, white line). Maintaining fixation on the dark but covertly shifting attention into the white periphery also affects the constriction via attention-related modulation of the PLR (i.e., resulting in small negative slopes; see Figure 7A, white line), but to a smaller extent (Binda, Pereverzeva, & Murray, 2013; Joshi & Gold, 2020; Mathôt, Van der Linden, Grainger, & Vitu, 2013). To isolate the pupil size change purely induced by the overt and covert attentional strategy, we finally calculated the difference between the white and the black slopes. With this white–black operation, we adjusted the slopes of the pupil size change for the influence of cognitive load.

### Statistical analysis

Statistics were calculated using IBM SPSS (version 25). All analyses of variance reported here were calculated as repeated measurement ones. If data violated the assumption of sphericity, a Greenhouse–Geisser correction was applied. Effect sizes are reported using partial eta-squared (*η_p_^2^*) for all parametric tests. Post hoc analyses were always done with Bonferroni corrected α-values. The results of the post hoc comparisons are denoted within the corresponding figures reflecting conventional significance levels (unmarked are not significant; ∗*p* < 0.05; ∗∗*p* < 0.01; ∗∗∗*p* < 0.001). All correlations were calculated using Spearman's rank correlation (rank coefficient = *r_s_*, two-tailed). Error bars in all figures indicate standard error of the mean.

## Results

### Task performance: Corsi span

A Corsi subtrial was counted as correct, if the positions of all targets were recalled in the correct order. The number of correct subtrials (0–4) in terms of condition and sequence length is provided for each participant in Supplementary Table S1.

**Figure 3.**
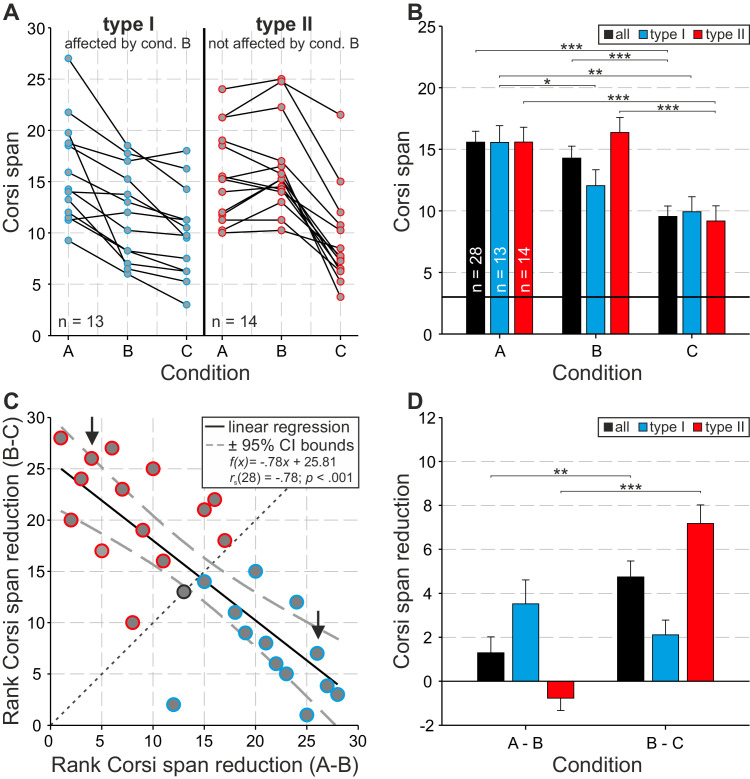
Absolute Corsi span and Corsi span reduction classifying type I (i.e., affected by condition B, preferring the overt strategy; blue) and II (i.e., not affected by condition B, preferring the covert strategy; red). (**A**) Individual Corsi span of all three conditions (A, without any restriction; B, restricted overt; and C, restricted overt and covert strategy) separately plotted for type I (*n* = 13) and type II (*n* = 14) participants. (**B**) Group analysis of Corsi span for all conditions and over type I, type II, and all (black) participants. (**C**) Linear regression (black line) together with correlation estimates between the individual ranks (1–28) of Corsi span reduction A–B and B–C (note the black circle denotes a neutral participant). The ±95% confidence bounds are depicted as gray, dashed curves. The diagonal separates participants into type I versus type II. The black arrows indicate the two participants, exemplary chosen for showing typical scanpaths (cf., Figure 4). (**D**) Group analysis of Corsi span reduction A–B and B–C for type I, type II, and all (black) participants.

Based on these values, the absolute Corsi span was calculated for each participant and condition (Figure 3A). Here, Corsi span values show a high variability between participants for each condition. Conducting a one-factorial analysis of variance revealed a significant effect of condition on overall Corsi span. *F*(2,52) = 36.15, *p* < 0.001, *η_p_^2^* = 0.6 (cf., Figure 3B). Interestingly, two general patterns regarding the change of Corsi span (i.e., Corsi span reduction) between the three conditions became obvious. A type I pattern is defined by a large decrease in Corsi span between condition A and B and no substantial difference between B and C (Figures 3A–D, blue symbols and bars). Participants of the type II revealed the opposite pattern. Here, no significant difference was observable between condition A and B, but a large decrease was found between B and C (Figures 3A–D, red symbols and bars). Again, condition had a significant effect on Corsi span for both types of pattern, type I: *F*(2,24) = 14.92, *p* < 0.001, *η_p_^2^* = 0.58; type II: *F*(2,26) = 48.19, *p* < 0.001, *η_p_^2^* = 0.79. The classification into type Is and II is based on the strong (negative) correlation between the ranks of Corsi reduction A − B and B – C, *r_s_* = − 0.78, *n* = 28, *p* < 0.001 (Figure 3C), indicating that participants with a high Corsi span reduction between conditions A and B show a rather similar span for B and C, and vice versa (see also Figure 3D). Consequently, all participants below the diagonal (see Figure 3C) belong to type I and all above it to type II (note that one participant could not be classified).

### Overt and covert strategy: Exploration index

**Figure 4.**
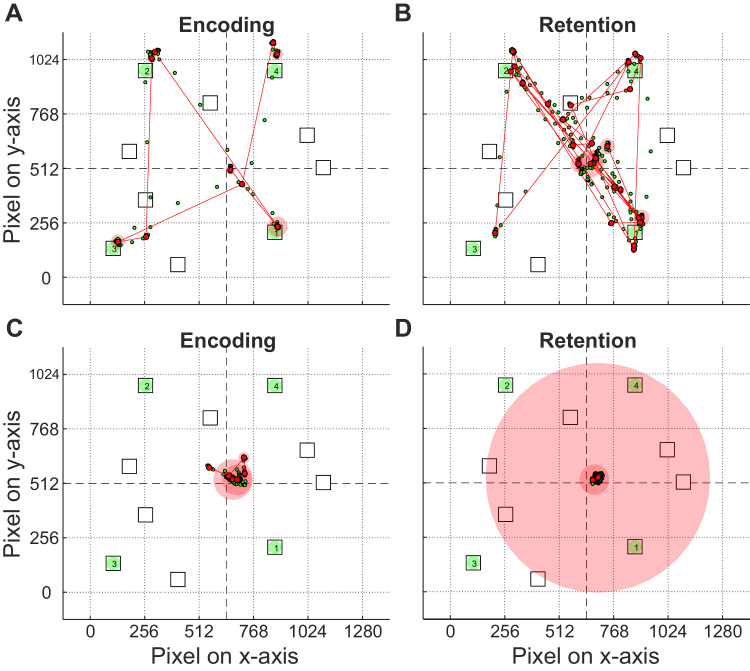
Examples of scanpaths of an overt (**A** and **B**) and a covert participant (**C** and **D**) for the encoding (left) and the retention phase (right) of condition A (without any restriction). The corresponding participants were marked with black arrows in Figures 3C and 6A). Corsi sequence with a sequence length of 4. The numbers in the targets (green) indicate their temporal order during presentation (note that targets were not shown in the retention phase; cf., Figure 1). Eye tracking raw data are displayed as small green dots, fixations as red dots, and saccadic periods as red lines between the fixations. Duration of fixations is indicated by light red discs, where their size corresponds with duration.

A new and intriguing result in terms of eye movement patterns and their meaning regarding encoding and retention of Corsi sequences was the finding about a high inter-individual variability, but the intra-individual stability of attention strategies. This finding is exemplary shown in the scanpaths of two participants (marked with black arrows in Figure 3C) using either overt or covert strategies in condition A (see Figure 4).

The overt participant (Figure 4, top) showed a general exploration pattern where fixations closely correspond with the target positions. The averaged exploration index over all sequence lengths of this participant was 0.9 (Figure 6A, right arrow). Such a pattern was also observable in the retention phase, where the targets were hidden. Contrary, nearly all fixations of the covert participant for the short sequence length 4 were found directed to the center of the display (Figure 4, bottom), showing no overt shifts of attention. The averaged exploration index over all sequence lengths of this participant was with 0.45 quite small (cf., Figure 6A, left arrow).

To quantify and normalize the extent of overt gaze shifts for each participant, the exploration index was calculated for all conditions (encoding and retention phase) involving sequence lengths of 3 to 5. Hence, the exploration index ranged from 0 (all fixations were located at the center; minimum looking at items) to 1 (the positions of the fixations were identical with the targets; maximum looking at items).

**Figure 5.**
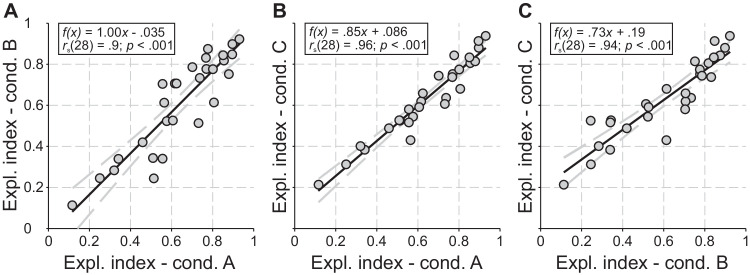
Stability of exploration index within the encoding phase between conditions A (without any restriction), B (restricted overt strategy), and C (restricted overt and covert strategy). Linear regressions (black line) together with correlation estimates between the individual exploration indices of (**A**) condition A versus B, (**B**) condition A versus C, and (**C**) condition B versus C. The ±95% confidence bounds are depicted as gray, dashed curves.

**Figure 6.**
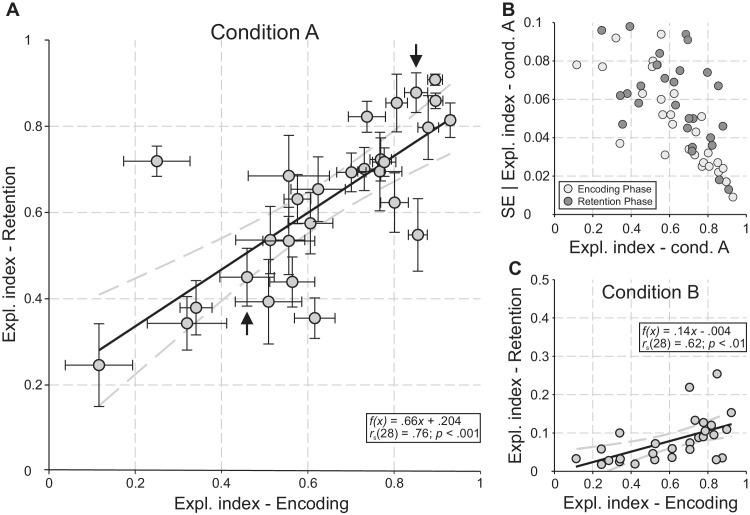
Stability of exploration index within conditions A (without any restriction) and B (restricted overt strategy). (**A**) Linear regression (black line) together with correlation estimates between the individual exploration indices of encoding versus retention of condition A. The black arrows indicate the two participants, exemplary chosen for showing typical scanpaths (cf., Figure 4). (**B**) Dependency of variability of the exploration index of condition A on the variance of exploration for encoding (light gray dots) and retention (dark gray dots). (**C**) Linear regression (black line) together with correlation estimates between the individual exploration indices of encoding versus retention of condition B. The ±95% confidence bounds are depicted as gray, dashed curves.

Interestingly, participants showed a very robust pattern of exploration both within the encoding phase for all conditions (Figures 5A–C) and within condition A for encoding and retention (Figure 6A). In other words, a participant with a high degree of overt attention during encoding of condition A will preserve this strategy also in the encoding phases of condition B and C (cf., high correlation values in Figures 5A–C), as well as in the retention phase of condition A (cf., high correlation value in Figure 6A).

The variability of exploration index for each participant was calculated over all 12 subtrials (3 sequence lengths × 4 subtrials each) of condition A, separately for encoding and retention. The correlation in Figure 6B shows that participants with higher exploration indices also maintain such behavior much more reliably compared with rather covert participants. Another surprising result is the correlation between the exploration index of the encoding and retention phase, which can also be found in condition B (Figure 6C). Because participants were prevented from performing regular eye movements during the retention phase, no such correlation should be expected. However, it seems that, within the tolerance window (cf., Figure 1), meaningful eye movements (scaling with the corresponding exploration tendency during encoding) were nevertheless executed, but with a smaller amplitude.

### Overt and covert exploration and cognitive load: (Slope of) pupil size change

**Figure 7.**
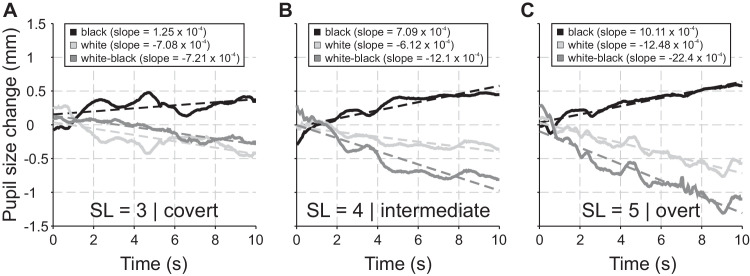
Change of pupil size during the retention phase of condition B (restricted overt strategy) shown for (**A**) a covert participant and a sequence length (SL) of 3, (**B**) another intermediate participant and a SL of 4, and (**C**) an overt participant and a SL of 5. Baseline corrected and smoothed data of pupil sizes are depicted as solid lines and their regressions as dashed lines (black with black lines, white with light gray lines, and white–black with dark gray lines). The slopes of the regressions for black, white, and white–black are provided in the legends.

A further promising measure concerning the distinction between overt and covert exploration together with the quantification of cognitive load is the change of pupil size. Here, the only relevant period of the experiment was the 10-second retention phase of condition B, because participants were instructed to maintain fixation in the center of the screen. Analyzing the pupil size change of each participant for the subtrials with a black center and background (black) provides a quantifier of cognitive load, because changes in pupil size owing to varying brightness can be excluded. In black subtrials, an increased cognitive load accounts for higher positive slopes. Positive slopes increase with longer sequences (cf., Figures 7A–C, black lines). Because black only quantifies the cognitive load (without the impact of rehearsal strategy), solely the sequence length or task complexity influences the traces here. Changes in the slopes for subtrials with a black center and white background (white’ may be caused by the combined influence of cognitive load and PLR. The PLR is the rapid and relatively strong constriction/dilation of the pupil diameter in response to an increase/decrease in light intensity (Clarke et al., 2003; Joshi & Gold, 2020; Loewenfeld & Lowenstein, 1993). Finally, subtracting the black slopes from the white ones will only leave negative slopes (cf., Figures 7A–C, white–black lines) owing to overt and covert shifts of attention. Because white–black was adjusted for the influence of cognitive load, solely the type of strategy (covert, intermediate, and overt) influences the traces here (for a detailed description of this framework, see Pupil size change).

**Figure 8.**
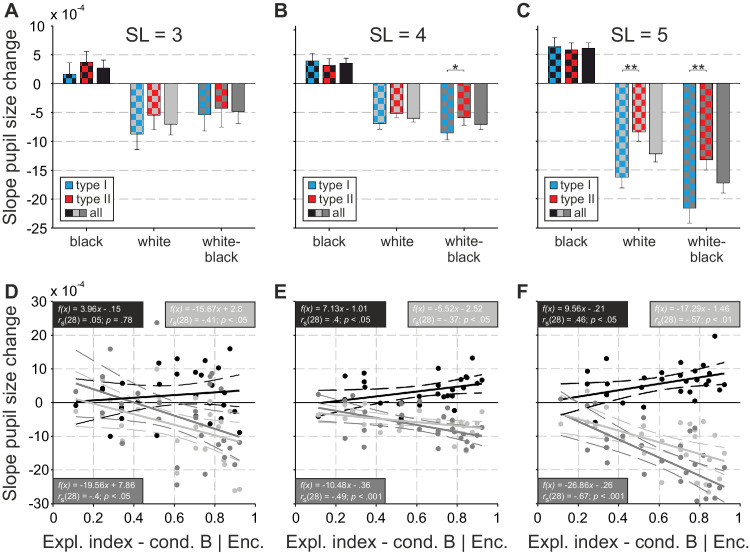
Slope of the pupil size change during the retention phase of condition B (restricted overt strategy). Averaged values for type I (i.e., preferring the overt strategy; blue), type II (i.e., preferring the covert strategy; red), and all (gray) participants and for black (black), white (light gray), and white–black (dark gray) traces are displayed for sequence lengths (SLs) od 3, 4, and 5 (**A**, **B**, and **C**, respectively). Correlations between individual exploration indices (encoding phase of condition B) and the slopes of pupil size changes are depicted for SLs of 3, 4, and 5 (**D**, **E**, and **F**, respectively). The black, white, and white–black data (dots), their regressions (straight lines) together with the ±95% confidence bounds (dashed curves) are shown together with the statistics.

Figure 7 shows representative traces of the change of pupil size over the 10 seconds of the retention phase (condition B) for the black, the white, and the subtraction of white − black analyses. Here, traces from a participant with a rather covert strategy and sequence length 3 (Figure 7A), another one with an intermediate strategy and sequence length 4 (Figure 7B), and a participant with a rather overt strategy and sequence length 5 (Figure 7C) are shown. The positive slopes of the black regressions (black lines in Figure 7) rise from sequence length 3 to 5, showing the increasing demand on cognitive resources, that is, working memory load. The negative slopes of the white–black lines exclusively quantify the effect of the PLR (adjusted for cognitive load) and they become more negative with increasing sequence length, which reflects the maximum extent of pupil constriction owing to shifts of attention to bright areas for the overt participant (Figure 7C, dark gray line). The group analysis regarding the slope of the pupil size change calculated for all participants and separately for those belonging to type I or II (Figure 3) is plotted in Figures 8A–C. The sequence length was significantly effecting the increase of the slopes of the black traces for the group of all participants, *F*(2,52) = 3.4, *p* < 0.05, *η_p_^2^* = 0.12; post hoc testing revealed a significant difference of *p* < 0.05 between sequence lengths 4 and 5. This increase reflects the generally higher memory load for longer sequences. There was no significant difference found for the black slopes between type I and type II participants at any sequence length, which shows that the memory load was similar for both types. The sequence length was also significantly affecting the decrease of the slopes of the white traces for the group of all participants, *F*(2,52) = 7.18, *p* < 0.01, *η_p_^2^* = 0.22; post hoc testing revealed a significant difference of *p* < 0.05 between sequence lengths 3 and 5 and of *p* < 0.001 between sequence lengths 4 and 5. Here, the combined effects of memory load and PLR lead to an increased negativity of the slopes. There was no significant difference found for the white slopes between type I and type II participants for sequence lengths 3 and 4; however, for sequence length 5, there was (Figure 8C). Likewise, sequence length was significantly affecting the decrease of the slopes of the white–black traces for the group of all participants, *F*(2,52) = 21.37, *p* < 0.001, *η_p_^2^* = 0.45; post hoc testing revealed a significant difference of *p* < 0.001 between sequence lengths 3 and 5 and of *p* < 0.001 between sequence lengths 4 and 5. The white–black traces were used here to show the change in pupil size caused purely by the overt and covert attention strategies. There was no significant difference found for the white–black slopes between type I and type II participants for sequence length 3; however, for sequence lengths 4 and 5 there was (cf., Figures 8B and C), indicating that the PLR was stronger in type I participants when the task difficulty was high.

To understand the pupil changes further, their individual slopes (concerning the black, white, and white–black traces) were correlated with the exploration index of the encoding phase of condition B (see Figures 8D–F). Here, except the blackslopes for sequence length 3, all other correlations tested as significant (cf., statistic estimates provided in the plots). For all sequence lengths, the more (overt) explorative a participant was, the higher was the slope of his black traces. It also shows that the larger the exploration index was the more negative was the slope of the participants’ white–black traces.

## Discussion

The present study investigated and quantified the employment of overt and covert attention as a strategy for encoding and retaining spatiotemporal information in a Corsi (block-tapping) task. We hereby aimed to disentangle the effects of VSWM, attention, and oculomotor rehearsal during the retention phase by systematically impeding eye movements and/or VSWM during three different task conditions. While in condition A no restrictions were imposed, the overt strategy (i.e., the execution of eye movements) was restricted in condition B and both strategies, overt and covert were restricted in condition C. In the following, we discuss the different influences of the three conditions on Corsi span leading to the distinction between type I versus type II participants (see Corsi performance). We then link Corsi performance first to the exploration index and second to pupil size (change) and discuss the effects of Corsi span reduction in view of overt and covert strategies (see 4.2. Explaining Corsi performance with exploration index and pupil size change). Finally, overt and covert attention-based strategies are analyzed and discussed in a larger context to generate hypotheses and questions for future research (see Discussing rehearsal strategies).

### Corsi performance

The Corsi task performance was analyzed using the Corsi span measure. Considering sequence length, the Corsi span quantifies the level of difficulty or complexity at which a participant was still able to perform the task correctly (cf., Weicker, Hudl, & Thöne-Otto, 2017). Additionally, the Corsi span may provide a measure of VSWM capacity in spatial tasks, that is, the amount of spatial memory that can be recruited for a particular task (Berch, Krikorian, & Huha, 1998). In our study, the Corsi span was calculated for each participant and condition. As expected, the overall Corsi span decreased significantly from condition A to C as the task difficulty increased. The relation of the individual reductions in Corsi span between conditions A–B and B–C of all participants shows a highly correlated and meaningful pattern (see Figure 3C). Here, participants spread a continuum from a high reduction A–B (with almost no reduction B–C) to high reduction B–C (with almost no reduction A–B). Thus, approximately one-half the participants (13 of 28) are impaired when oculomotor rehearsal is disallowed (classified as type I), whereas the other one-half (14 of 28) only shows a significant performance drop (classified as type II) when an additional memory task was given during the retention phase (see Figures 3B and 3D). Although both strategy types achieve similar levels of performance in conditions A and C, the difference between type I and II is greatest in condition B (see Figure 3B). It turns out that conditions A and C limit the Corsi performance in the same way for all participants (and strategy types), ranging from 15.6 ± 0.9 (condition A) to 9.6 ± 0.9 (condition C). Note that, with values around 10, neither type I nor II reached the lowest possible Corsi span of 3 in condition C, that is, all participants complete at least one subtrial correctly up to sequence length 5 (see Supplementary Table S1).

The aim of condition C was to prevent attentional shifts (overt and covert) during the retention phase by using a secondary task that directed attention to the center of the screen. There is the possibility that the additional task interferes with the Corsi task and thus does not allow a distinction between the two types I and II. Our results do not indicate such an effective interference, as type I (overt) participants do not show a significant decrease in their Corsi performance between conditions B and C (see Figure 3B). In addition, with values around 10, neither type I nor II reached the lowest possible Corsi span of 3 in condition C, that it, all participants completed at least one subtrial correctly up to sequence length of 5 (see Supplementary Table S1). In line with this, Rudkin, Pearson, & Logie (2007) showed that a Corsi task was more impaired by a central executive secondary task (e.g., random number generation) than by a simultaneous spatial task (i.e., matrix pattern task), which we used in our study.

In the Corsi task, rehearsal is mainly used to retrace the spatiotemporal sequences in the correct order during retention. If such rehearsal is disturbed or prevented, the task performance drops significantly (Martin, Tapper, Gonzalez Leclerc, & Niechwiej-Szwedo, 2017; Pearson & Sahraie, 2003; Sdoia, Di Nocera, & Ferlazzo, 2019). The Corsi spans of type II participants in conditions B indicate that they had no difficulties with this condition. Here, a reasonable strategy would be to use covert attention as a means of rehearsing the Corsi sequences. Such behavioral strategy has already been described in Corsi tasks (Godijn & Theeuwes, 2012; Patt et al., 2014; Tremblay et al., 2006). The strong decrease in performance from condition B to C is due to the fact that the use of both possible strategies is restricted in condition C. Type I participants may have the opportunity to overcome the constraint in condition B by performing small-scaled overt shifts of attention (within the tolerance window of the fixation stimulus; cf., Figure 6C). However, please refer to the following sections for a more detailed discussion of the different rehearsal strategies.

### Explaining Corsi performance with exploration index and pupil size change

To shed more light on the factors that determine Corsi performance and to characterize participants' strategy types I and II in more detail, the two eye-specific measures, exploration index (i.e., extent of eye movements) and pupil size change (i.e., dilation/constriction of pupil), are discussed in the following.

The exploration index (ranging from 0 to 1) was introduced to quantify the extent of meaningful eye movements, that is, fixations on items or targets in the Corsi sequences. The exploration index was maximal if, for each item in a given sequence, at least one distinct fixation matched. The index was minimal if, on the other hand, all fixations were located on the fixation cross, that is, maximally far away from the targets (in direction of the center). Furthermore, the higher the index the more likely a participant showed an overt strategy (use of eye movement) and the lower the index the more likely a participant showed a covert strategy (use of attentional shifts or other).

An interesting finding is that all participants showed a stable choice of their overt versus covert strategy between encoding and retention (cf., Figures 4 and 6A–B) and between all conditions (A vs. B; A vs. C; B vs. C) of the retention phase (cf., Figure 5). This strong intra-individual stability is surprising, because no task constrains or instructions regarding any oculomotor behavior were given. This result means that the participants' strategy choice was caused by an intrinsic property. Our findings show that the majority of participants chose a rather overt strategy, i.e., at least 20 of 28 of them were quantified with an exploration index above 0.5. Even when it represents a clear disadvantage in the corresponding condition, participants tend to stick with their preferred strategy, which was chosen when there were no constraints. Interestingly, participants who preferred an overt strategy still performed micro-scaled eye movements (small movements within the tolerance window) during the retention period of condition B (when eye movements were suppressed owing to forced central fixation) and therefore showed increased exploration index values (cf., Figure 6C). Participants were consciously aware that they were supposed to maintain a fixed gaze during this period of the experiment. Hence, the compulsive execution of eye movements might indicate a subconscious urge to comply with the preferred overt retention strategy, even when external circumstances prevent those. This observation is in line with the finding of high intra-individual stability of strategy choice across phases, conditions and difficulty levels (involving sequence lengths from 3 to 5) and consistent with the suggestion that information is maintained in VSWM in the same way it was encoded, that is, in its original representation (D'Esposito, 2007; Patt et al., 2014).

One might note that participants who do not show eye movements in condition A prefer this covert strategy because they were primed by the instructions in conditions B and C (where eye movements were prevented). However, when the experimenter asked about the strategies or noticeable problems or features concerning the Corsi task at the end of the experiment, no participant reported any influence of the instruction. Although the behavior of covert shifts of attention during encoding and retention seems surprising, this strategy was also found to be a common pattern in other studies investigating rehearsal and memory for serially presented spatial locations (Godijn & Theeuwes, 2012; Lilienthal, 2018; Patt et al., 2014; Smyth & Scholey, 1994; Tremblay et al., 2006). We also found in previous Corsi experiments without conditions that prevented the overt strategy that participants “voluntarily” (i.e., without instructions for a specific strategy) and reliably used covert attention. Overall, we therefore rule out priming of the attention-based strategy by the instructions with high probability.

The distinction between overt and covert strategies found in our Corsi experiment was further supported by the advanced measure of pupil size change. Again, regarding this measure, the retention phase of condition B was the only relevant period of the experiment (as only there participants had to keep fixation in the center of the screen). Our data show clearly that pupil size was influenced by both sequence length (3 to 5; cf., Figures 7 and 8A–C) and strategy type (I and II; cf., Figures 8A–C). Furthermore, we found meaningful correlations between the exploration index and the slope of pupil size change (cf., Figures 8D–F). The pupil behavior during the black subtrials reflects merely cognitive load. Here, the change of pupil size over time shows an increase for all strategy types and sequence lengths. However, the slope was strongest at sequence length 5 and not influenced by the type of strategy (see Figures 8A–C). During white subtrials, the pupil size was influenced by cognitive load together with the PLR. Here, the (slope of) change of pupil size can be used to discriminate between oculomotor strategies. When a participant tried to maintain the overt strategy and performed micro-scaled eye movements, the pupil constricted more than when a covert participant fixated strictly on the fixation cross that was always presented on a dark background (Figure 1). In the first case, the white area surrounding the black center affects the constriction directly since the light conditions change (i.e., PLR). In the latter case, covert shifts of attention into the white area also affects the constriction via attention-related modulation of the PLR, but to a smaller extent (Binda et al., 2013; Mathôt et al., 2013). To isolate the pupil size change purely induced by the overt and covert attentional strategy, we finally calculated white–black, that is, the difference between the white and the black slopes. With this operation, we show that type I versus II participants match with overt versus covert (rehearsal) strategies. This correspondence is clearest for the sequence length 5 (cf., Figure 8C). Furthermore, we show significant correlations between an increasing exploration index and decreasing slopes of the pupil size change in white–black subtrials (see Figures 8D–F). These correlations link the exploration index measure to strategy type I versus II (at least for the retention phase of condition B) and we can finally argue that type I participants preferably use the overt strategy whereas type II participants preferably use the covert one.

### Discussing rehearsal strategies

VSWM is limited in both capacity (e.g., Cowan, 2010; Miller, 1956) and duration (e.g., Farrell et al., 2016). If we need to maintain information about the spatial configurations of objects surrounding us, an active process of rehearsal is used to counteract time-based forgetting (Awh et al., 2006; Godijn & Theeuwes, 2012; McAteer, McGregor, & Smith, 2022; Souza et al., 2020; Theeuwes et al., 2009). In terms of maintaining visuospatial information, two principles are described: attention-based (exogenous) and or mental-based (endogenous) retention (Awh et al., 1998; Cisek & Kalaska, 2004; Lilienthal et al., 2018; Lilienthal, 2018; Souza et al., 2020). Attention-based means that, for the rehearsal of to-be-remembered locations, participants can use overt (with eye/gaze movements) or covert attentional shifts (without execution of spatially guided movements). In addition to overt and covert attention, the involvement of higher-order and rather executive cognitive processes during spatial tasks is suggested. In mental-based retention, the role of imagery as a tool for visuospatial imagination is discussed in the literature (cf., Patt et al., 2014; Pearson, 2019; Savaki & Raos, 2019). Participants in our experiments were forced and instructed to keep their eyes open at all times to enable eye-tracking at each phase of the task. In addition, in the retention phase of all conditions, visual attention was directed overtly or covertly to the squares of the respective sequence to facilitate attention-based rehearsal (cf., Lilienthal et al., 2016; Souza et al., 2020). This is evident from the white–black slopes of the pupil size in Figures 8A–C, which quantify the increasing use of the attention-based strategy, where pupils constrict more as the complexity of the task increases owing to PLR. Taking these arguments into account, we can largely exclude imagination as a rehearsal strategy in our study because imagery primarily occurs in the absence of visual input, for example, in darkness or with eyes shut.

In our study, we revealed attention-based retention (i.e., both overt and covert shifts of attention) as an underlying mechanism for the rehearsal of Corsi sequences. The eye exploration of the participants show clearly that there is not a discrete or binary distribution between complete overt (exploration index = 1) and complete covert (exploration index = 0) attention. Rather, we found a range or continuum between overt and covert strategy (see Figures 5A–C and 6A). Intermediate values of the exploration index may occur when items are explored to a smaller extend. Interestingly, participants with a greater propensity for overt exploration during encoding in condition B (where they should keep the gaze on the center), also showed this strategy, but to a lesser extent, during retention. Such micro-scale scanning was possible owing to the size of the tolerance window (cf., dashed square in Figure 1) and is consistent with findings that microsaccades can be an overt measure of covert attentional shifts and operate on the same oculomotor system (Hafed & Clark, 2002). Another explanation for intermediate values of the exploration index could result from the variable use of the overt strategy either with respect to the items of a given sequence, for example, skipping an item during retracing (Rinaldi, Brugger, Bockisch, Bertolini, & Girelli, 2015) or with respect to the (sub)trials. Since our method of quantifying the exploration index is rather conservative, we cannot completely rule out any of the above possibilities.

#### Intra-individual stability of rehearsal

With the study, we intended to answer the question of the stability of the rehearsal strategy chosen individually by each participant. Our data show clearly that participants are highly stable in their choice of the selected overt versus covert strategy (i.e., intra-individual stability) with respect to different lengths of sequences (cf., Figure 8) and the phases (cf., Figure 6) as well as the condition (cf., Figure 5) of the Corsi task. The following question should then be whether one of the two strategies offers a performance benefit in the context of the Corsi task. Here, our data show that the preferred strategy is not decisive for the performance achieved in the task, that is, both type I (overt) and type II (covert) participants achieved the same Corsi span in condition A and C. Also Godijn and Theeuwes (2012) showed that both strategies are equivalent in terms of encoding as well as maintaining the Corsi information and further, information is maintained in VSWM in the same way it was encoded, i.e., in its original representation (D'Esposito, 2007; Patt et al., 2014). In conclusion, we are able for the first time to i) classify participants into a strategy space with regard to visuospatial rehearsal and retention and ii) show the robustness of such intra-individual strategy selection across the entire Corsi task.

#### Rehearsal in a sensorimotor continuum

The standard model of VSWM assumes that its functions arise from the operation of specialized systems that act as buffers for the storage and manipulation of information. A current and alternative view holds that VSWM operates flexibly in a sensorimotor continuum through the coordinated recruitment of brain systems across attention that have evolved to accomplish sensory-, representation-, and action-related functions (Christophel et al., 2017; Fuster, 2022; Postle, 2006).

Within this current framework, it is proposed that prospective motor encoding is involved (Postle & D'Esposito, 2003; Postle, 2006) also to control for rehearsal processes. Here, vision-based, retinotopic coordinates are transformed into a motor plan in relation to the viewer's body. Locations of objects are than memorized by maintaining the motor plan that will eventually be used to act upon it. Here, premotor planning and execution activity is found to support rehearsal. This is suggested for subvocal articulatory rehearsal to promote maintenance of verbal information (Baddeley, 1986; Buchsbaum & D'Esposito, 2019; Jacquemot & Bachoud-Lévi, 2021), as well as for the oculomotor system to promote the retention of spatial information (Boon et al., 2019; Patt et al., 2014; Zimmer, 2008). It is further proposed that the representation and maintenance of spatial information not stem solely from oculomotor processes, but likely recruits a more general motor planning system (e.g., Postle, 2006). VSWM as cognitive function represents information in a sensorimotor continuum (ranging from detailed sensory information in sensory areas to abstract, amodal information in prefrontal areas) (e.g., Christophel et al., 2017; Christophel et al., 2018; Fuster & Bressler, 2012; Liu et al., 2020) and it represents to perform an action (behavior), whether it be oculomotor, verbal, or otherwise (Postle, 2006). Such representations may arise from the coordinated recruitment of perceptual processes (retrospective; e.g., attention-based retention) (Awh & Jonides, 2001; Awh et al., 2006) and motor (prospective; e.g., mental-based retention) (Cisek & Kalaska, 2004; Olivers & Roelfsema, 2020) in any stage of the sensorimotor continuum (Fuster, 2022). Here, attention together with persistent activity serve as the maintenance mechanism of sensory-action coupling (Boon et al., 2019; Olivers & Roelfsema, 2020; Zimmer, 2008). Given such a sensorimotor continuum, participants could choose a mental representation “in” VSWM (Postle, 2006) that satisfies both individual prerequisites and task requirements to perform adequately in the Corsi paradigm. Whether and to what extent such representations are used in addition or to support overt and covert rehearsal in the context of Corsi strategies needs to be investigated in further studies.

In the future, more work is needed to investigate the use of retention/rehearsal mechanisms within the framework of a sensorimotor continuum (Liu et al., 2020; Olivers & Roelfsema, 2020; Fuster, 2022). Such a framework should consider higher and more abstract functions (such as motor plans, imagination) in addition to attention-based ones. Presumably, applying a more comprehensive framework would help us better understand the intermediate rehearsal strategies we have identified in our study.

## Supplementary Material

Supplement 1
