# Supplementary material for: Inter-individual variability (but intra-individual stability) of overt versus covert rehearsal strategies in a digital Corsi task
Source: J Vis. 2024 Aug 1;24(8):2. doi: 10.1167/jov.24.8.2 (PMC11305427; doi:10.1167/jov.24.8.2)
Supplement: Supplement 1 [file jovi-24-8-2_s001.pdf]

Supplementary Material

Supplementary Table 1. Number of subtrials (0 to 4) correctly recalled for each of the three conditions (A - without any restriction; B - restricted overt; and C - restricted overt and covert strategy) and sequence lengths (SL 3 to 8) provided for each participant (ID 1 to 28).

ID	1	2	3	4	5	6	7	8	9	10	11	12	13	14	15	16	17	18	19	20	21	22	23	24	25	26	27	28
**A - SL 3**	4	4	4	4	4	4	4	4	4	4	3	4	4	4	4	4	3	3	4	4	4	4	4	4	4	4	4	4
**A - SL 4**	2	4	4	4	4	4	4	2	4	4	3	3	4	4	2	4	3	4	4	3	3	3	4	4	3	3	4	4
**A - SL 5**	2	3	2	3	4	0	3	2	4	3	4	3	2	4	1	2	1	3	1	1	2	2	4	3	3	2	1	2
**A - SL 6**	1	1	2	2	3	0	2	1	3	1	2	1	1	3	0	1	1	1	0	0	0	1	2	2	1	1	0	2
**A - SL 7**	0	2	2	3	1	0	1	0	2	0	2	0	0	1	0	0	0	0	0	0	0	0	1	1	0	0	0	0
**A - SL 8**	0	0	0	1	0	0	0	0	2	0	0	0	0	0	0	0	0	0	0	0	0	0	0	0	0	0	0	0
**B - SL 3**	3	4	3	4	3	4	4	4	4	4	4	4	2	3	4	4	4	4	4	4	4	4	3	4	4	4	4	2
**B - SL 4**	3	4	3	4	4	3	3	4	4	4	3	3	3	4	0	4	4	3	4	3	1	3	1	4	3	1	3	4
**B - SL 5**	0	2	2	4	3	1	3	3	3	2	3	1	3	3	0	3	1	2	3	2	1	2	0	4	1	0	1	2
**B - SL 6**	0	3	3	3	4	0	2	1	2	1	1	0	1	3	0	0	0	1	1	1	0	1	0	1	0	0	0	1
**B - SL 7**	0	0	1	2	1	0	0	0	1	0	2	0	1	3	0	0	0	0	0	0	0	0	0	1	0	0	1	1
**B - SL 8**	0	0	0	1	0	0	0	0	0	0	0	0	0	1	0	0	0	0	0	0	0	0	0	0	0	0	0	0
**C - SL 3**	2	4	3	3	3	3	1	3	4	4	3	1	3	4	0	2	3	4	3	3	3	2	3	4	3	3	3	2
**C - SL 4**	3	2	3	3	3	2	0	1	3	3	4	0	2	4	0	2	1	2	0	0	1	3	0	1	2	1	2	2
**C - SL 5**	0	3	2	3	3	1	2	1	3	1	3	0	0	4	0	2	0	2	0	2	0	3	0	2	2	0	2	0
**C - SL 6**	0	3	0	2	0	0	0	0	1	0	1	0	0	2	0	1	0	0	0	0	0	0	0	0	0	0	1	0
**C - SL 7**	0	1	0	0	0	0	0	0	0	0	1	0	0	2	0	0	0	0	0	0	0	0	0	0	0	0	0	0
**C - SL 8**	0	0	0	0	0	0	0	0	0	0	0	0	0	0	0	0	0	0	0	0	0	0	0	0	0	0	0	0

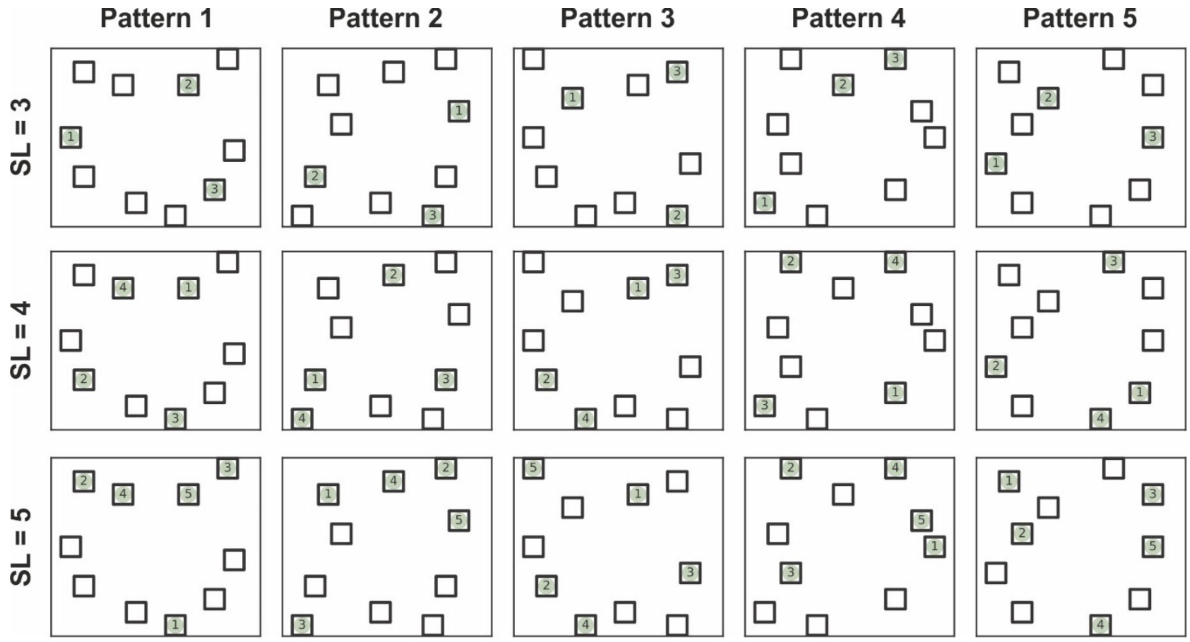

Supplementary Figure 1. Illustration of the five different default patterns (column 1 to 5) with examples of three different sequence lengths (SL; row 1 to 3) used in the study. Each pattern consists of ten squares (size of one square: 60 x 60 pixels, 2 x 2°), which are evenly distributed around the center. The overall spatiotemporal complexity of the sequences of items (green circles) was balanced concerning the number of path crossings, path lengths, and path angles. The numbers in the green circles indicate their respective order in the sequence.
